# A Versatile Route to Shape Polymer Nanoparticles by Deforming Nanoreactors Made from Magnetic Surfactants

**DOI:** 10.1002/anie.202422439

**Published:** 2025-03-26

**Authors:** Benjamin Botev, Stephan Siroky, Irene Morales, Sebastian Polarz

**Affiliations:** ^1^ Institute of Inorganic Chemistry Leibniz University Hannover Callinstrasse 9 30167 Hannover Germany; ^2^ Cluster of Excellence PhoenixD (Photonics, Optics and Engineering – Innovation Across Disciplines) Leibniz University Hannover 30167 Hannover Germany

**Keywords:** Anisotropy, Emulsion‐polymerization, Magnetic surfactants, Polymer nanoparticles, Sustainability

## Abstract

Directions are equivalent in an amorphous system, so anisotropy cannot emerge of its own accord, resulting in a gap for preparing polymer nanoparticles deviating from a spherical shape. Unlike inorganic nanocrystals for which faceting controls shape, polymers are not directly available as rod‐like particles, for instance. Here, we show a highly versatile, nontoxic, novel approach to break this paradigm and obtain polymer nanorods by emulsion polymerization using a unique surfactant comprising a magnetic head group. Surprisingly, even applying a weak magnetic field to the magnetic surfactant within an emulsion polymerization transforms diamagnetic polymers into rod‐like nanoparticles instead of their usual spherical shapes. The polarization in a magnetic field exerts a torque on the molecular structure, and as a result, the emulsion droplets deform. The method can be applied to different polymers such as polystyrene, polymethylmethacrylate, or polythiophene. The magnetic surfactant is recovered quantitatively and can be reused; one obtains metal‐free polymer particles, and the process is sustainable. The straightforward approach presented here will unlock several applications of these previously inaccessible polymer nanorods, particularly in the case of conducting polymers.

## Graphical abstract

Shaping up! Polymer nanoparticles are typically spherical, constrained by their structures. Now, a unique magnetic surfactant enables rod‐shaped polymer nanoparticles. Through emulsion polymerization and a magnetic field, the surfactant elongates nanoreactor droplets. Nanorods of polystyrene, polymethylmethacrylate, and polythiophene demonstrate versatility. This recyclable surfactant offers a sustainable path to polymer nanorods for many uses.

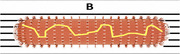

## Introduction

Polymeric nanoparticles (PNPs) are well‐established constituents of existing and emerging technologies. Despite them being made from abundant and nontoxic elements (C, H, N, S), a general substitution of functional inorganic nanocrystals is currently not possible. The predominant amorphous character of most conventional, atactic polymers makes it harder to obtain and stabilize ultrasmall PNPs (≪50 nm),^[^ ^1^ ^]^ and it is crucial that among the vast number of review articles,^[^ ^2^ ^]^ not one is dedicated to PNP systems characterized by inherent shape anisotropy. Instead, one finds numerous reports about composites of inorganic materials with macromolecular compounds, e.g., coating strategies of hard‐matter nanoparticles as a core.^[^ ^3^, ^4^ ^]^ A shape deviating from spherical is the rule for inorganic nanocrystals as their shape is determined by the energetic interplay of surfaces corresponding to lattice planes (*hkl* = Miller indices) and capping agents binding to them.^[^ ^5^ ^]^ As opposed to this, minimizing the surface‐to‐volume ratio, combined with the amorphous character, rationalizes why the known PNP systems almost exclusively exhibit a spherical morphology.

Only a few researchers could successfully prepare pure PNPs deviating significantly from a spherical shape, and if so, the particle formation process correlates to a crystallization process.^[^ ^6^ ^]^ Warren et al. described the fixation of liquid‐crystalline structures formed by macromolecular amphiphiles.^[^ ^7^ ^]^ Fernandez‐Rico et al. presented an impressive work in which curvature was introduced by a post‐synthetic treatment with UV light.^[^ ^8^ ^]^ Identifying a direct synthetic route that can deliver practically a wide range of polymers in the form of nanoparticles possessing an anisotropic morphology is a significant advancement, and it would open numerous perspectives for a new class of polymer‐nanoparticle‐based materials.^[^ ^9^ ^]^

Among the different polymerization techniques, emulsion‐based methodologies are in a prime spot. Emulsion polymerization is a process where monomers are polymerized in an aqueous medium to form polymer particles dispersed in water. This technique uses surfactants to create stable emulsions by forming micelles, which encapsulate the monomers and facilitate their polymerization within these droplets. The surfactants help to control the particle size and distribution, forming a stable colloidal suspension of polymer particles. A special class of designer surfactants that could be realized recently is equipped with a magnetic moment (MagSurfs).^[^ ^10^, ^11^, ^12^ ^]^ We design new surfactants with a head group containing a paramagnetic metal ion coordinated by a macrocyclic ligand.^[^ ^13^, ^14^ ^]^ We could already show that one can deform micellar aggregates by an external magnetic field. The concept of the present paper focuses on utilizing MagSurfs as emulsification agents in an external magnetic field to polymerize a variety of monomers. While styrene serves as our model monomer, we highlight the versatility of the method with also applying it to methylmethacrylate and thiophene. The polymer nanorods are isolated as the final product, free of MagSurf and thus metal‐free. Importantly, the MagSurf remains intact and can be recycled, enabling repeated polymerization processes with the same reused MagSurf, which emphasizes the sustainability and eco‐friendly aspect of our approach. We demonstrate the direct formation of anisotropic polymer‐based nanoparticles with distinct shapes controlled by magnetic field strength, within a simple implementation, no post‐synthetic procedure needed. Therefore, we show a clear alternative to established techniques, offering a simple and more sustainable solution for polymer‐based nanoparticle production.

## Results and Discussion

### The Magnetic Surfactant

The organic ligand 4,7,10‐tetraazacyclododecane‐1,4,7,10‐tetraacetic‐acid (DOTA) attached to a single octadecane chain (C_18_DOTA) as the nonpolar part of the surfactant was prepared by a modified synthetic protocol (see the experimental part in the Schemes S1–S3).^[^ ^14^, ^15^, ^16^ ^]^ We have selected Mn^2+^ as a metal ion (Scheme S4) because it delivers the high‐spin complex and possesses no hazardous properties to humans or the environment. Data for the characterization of the MagSurf C_18_DOTA‐Mn can be found in the Figures S1–S3. For instance, electron paramagnetic resonance (EPR) spectroscopy of the surfactant in solution confirms the paramagnetic character. One sees the sixfold peak, which comes from hyperfine interaction of the 5/2 spin system with the nucleus (*I* = 5/2) (Figure S3).^[^ ^17^ ^]^ Because the self‐assembly process of C_18_DOTA‐Mn in water has been reported before,^[^ ^14^ ^]^ it is not described here again.

### Emulsion‐Polymerization of Styrene in MagSurf Stabilized Droplets

**Figure 1:**
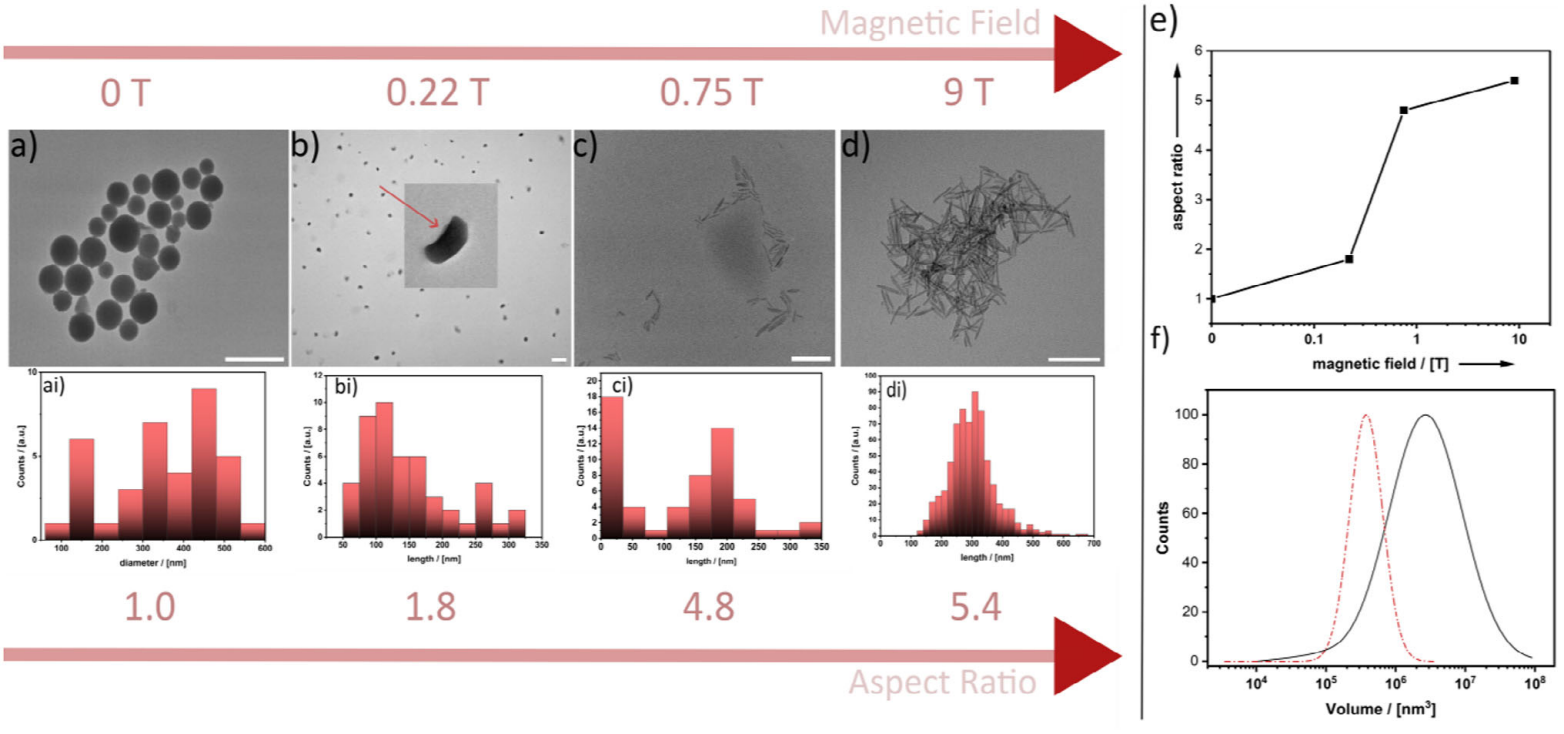
TEM micrographs (scalebar = 100 nm) and the particle‐length distribution of polystyrene latices obtained from polymerization in MagSurf stabilized droplets at a different, external B‐field a–d). The distribution of the diameters and lengths of the respective particles is shown in ai‐di (ai: *σ* = 100.843; bi: *σ* = 41.634; ci: *σ* = 84.213; di: *σ* = 79.414.) The aspect ratio is a nonlinear function of the magnetic field strength e). Comparison of the volume of the spherical polymer particles (black curve) and 9 T (red dotted line) assuming a cylindrical shape f).

We used the MagSurf to form a stable oil‐in‐water emulsion (20% styrene:80% H_2_O). A photograph of the resulting emulsion is shown in Figure S4. The droplet size ranges from 50 to 150 nm according to dynamic light scattering (DLS). The monomer's viscosity suits the proposed method, with lower viscosity monomers improving droplet formation in the emulsion. In contrast, highly viscous monomers impede diffusion within the monomer droplets, consequently hindering the reaction rate.^[^ ^18^ ^]^ Excessive viscosity affects molecular movement at the interfaces. Reaction kinetics (heat), mass, and instantaneous transfer within the polymerization reactor are crucially impacted by viscosity changes.^[^ ^19^ ^]^ The concentration was meticulously chosen; we maintained the monomer proportion lower than the water proportion in the emulsion to prevent agglomeration effects. Conducting the reaction at room temperature enhances emulsion stability and prevents Mn^2^⁺ oxidation, ensuring stable coordination with the head's ─OH groups. The emulsion was then exposed to a magnetic field for 30 min. Ethanol was added, leading to the polymer's precipitation and the polymerization process is ended. The precipitate was separated by centrifugation. ^1^H‐NMR spectroscopy of the compound dissolved in deuterochloroform (CDCl_3_) proves that polystyrene (PS) has been obtained (Figure S5). The samples were investigated by transmission electron microscopy (TEM). Without a magnetic field (*B* = 0 T), spherical latex particles are obtained, as shown in Figure 1a.

The particle‐size distribution is polydisperse and ranges from 100 to 600 nm. The latter results are confirmed by DLS measurements, as shown in the Figure S5. The polymerization process was repeated, but the emulsion was placed inside the strongest magnetic field accessible to us (*B* = 9 T). One can see from Figure 1d that the shape of the polymeric particles is now highly anisotropic. The length of the nanorods ranges from 100 to 500 nm with a diameter below 10 nm. To confirm that the observed effect is caused by the MagSurf stabilizing the emulsion droplet, a reference experiment was performed using the “empty” C_18_DOTA. As expected, the diamagnetic, metal‐free C_18_DOTA delivers spherical polystyrene particles; see Figure S7. There is clear evidence that the magnetic force exerted on MagSurf is in some way responsible for the deformation of the polymer particles. Encouraged by the latter result, weaker magnetic fields were applied (*B* = 0.75 T; 0.22 T). It is important to note that such fields can be produced by conventional permanent magnets. Images showing the setup used for the application of the magnetic field are shown Figure S8. That neither electromagnets nor superconducting magnets are necessary is a significant advantage of the system presented here. TEM micrographs of the resulting samples are shown in Figure 1b,c. One can see that the aspect ratio, defined as the length of the particles divided by the diameter, is a nonlinear function of the external magnetic field (Figure 1e). At *B* = 0.22 T, the particles have begun to elongate and adopt a banana‐like shape. Because at *B* = 0.75 T, the maximum aspect ratio is nearly reached, one can conclude that a fine‐tuning of the particle morphology is possible at low fields in the range of 0.2–1 T.

### MagSurf Recycling and Reuse

**Figure 2:**
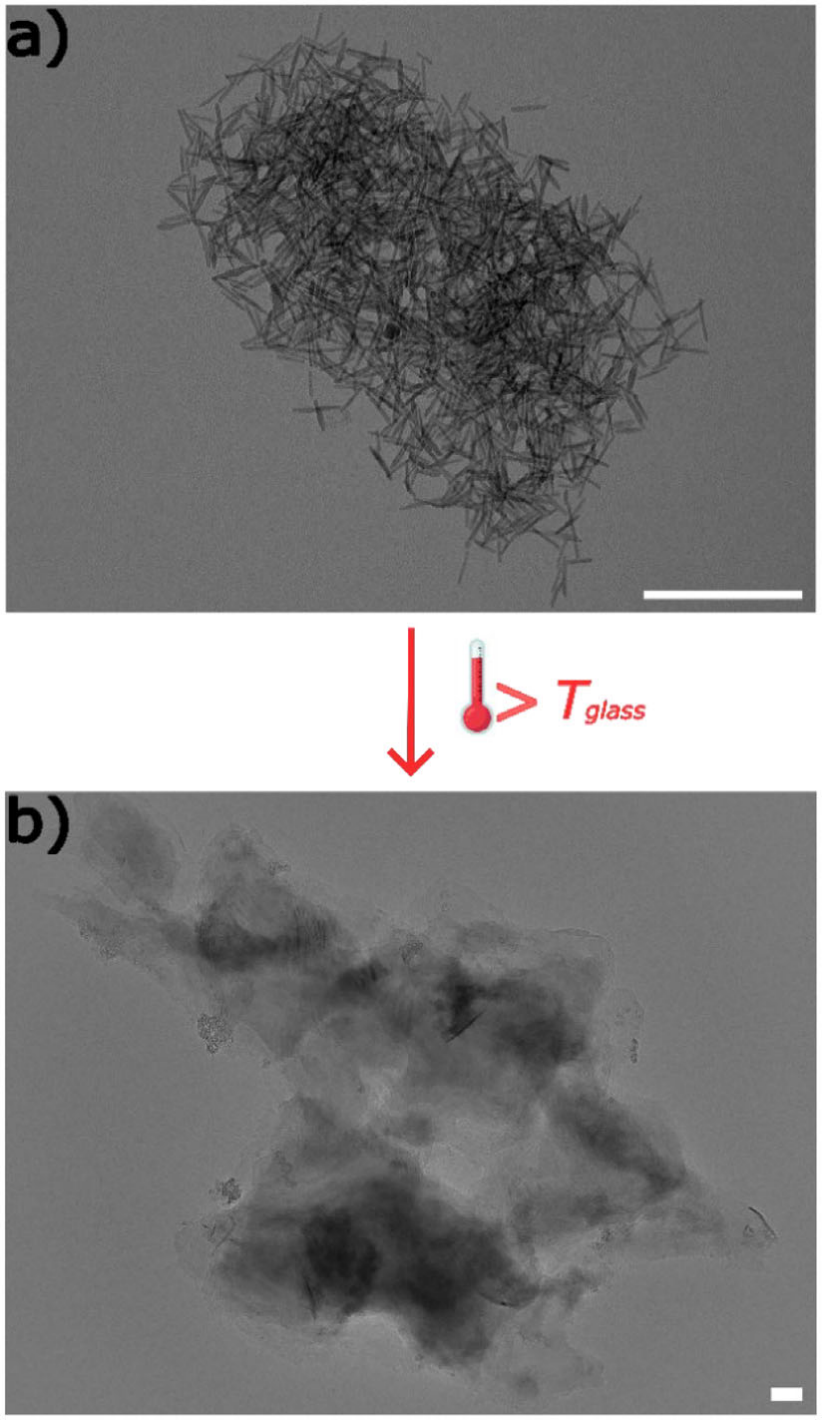
TEM micrographs a) of PS nanorods obtained by polymerization with reused MagSurf (scalebar = 500 nm); b) of a sample PS nanorod sample heated above the glass point (*T* > 100 °C; scalebar = 100 nm).

A highly important question is: Where is the surfactant located when the polymer synthesis is finished? For clarification, we have analyzed the precipitated polymer and the supernatant in more detail. Water was removed from the supernatant, and a solid was obtained. According to various analytical data shown in Figure S9, including Fourier‐transform infrared spectroscopy (FT‐IR), EPR, and thermogravimetric analysis (TGA), this solid is C_18_DOTA‐Mn. An important question is whether a fraction of MagSurf remains bound to the latex particles or could be removed quantitatively. A first indication that the surfactant has been removed quantitatively from the polymeric product is to see what happens when one heats the obtained nanorods above the glass transition temperature *T*_g_ (Figure 2b). As expected for pure polystyrene, the particles lose their shape and coalescent into a macroscopic polymer piece without any special structure. Next, we apply various analytical methods to prove that no surfactant remains in the polymer nanoparticles. ICP‐OES measurements of the centrifuged and washed polystyrene confirm that Mn^2+^ is virtually undetectable, with results below the detection limit. Also, in energy‐dispersive X‐ray spectroscopy (EDX) and EPR spectroscopy, there is no evidence of Mn^2+^ ions (see Figure S9d–f). The anisotropic particles consist of pure polymer. At the same time, the latter result means that we can quantitatively recover the intact MagSurf. For a proof of concept, the recovered surfactant was used to repeat the emulsion polymerization process in a magnetic field (Figure 2a). Obviously, PS nanorods can be prepared repeatedly. The recycling of MagSurf was repeated three times with same results, demonstrating its reusability.

### Particle Formation Mechanism

**Scheme 1:**
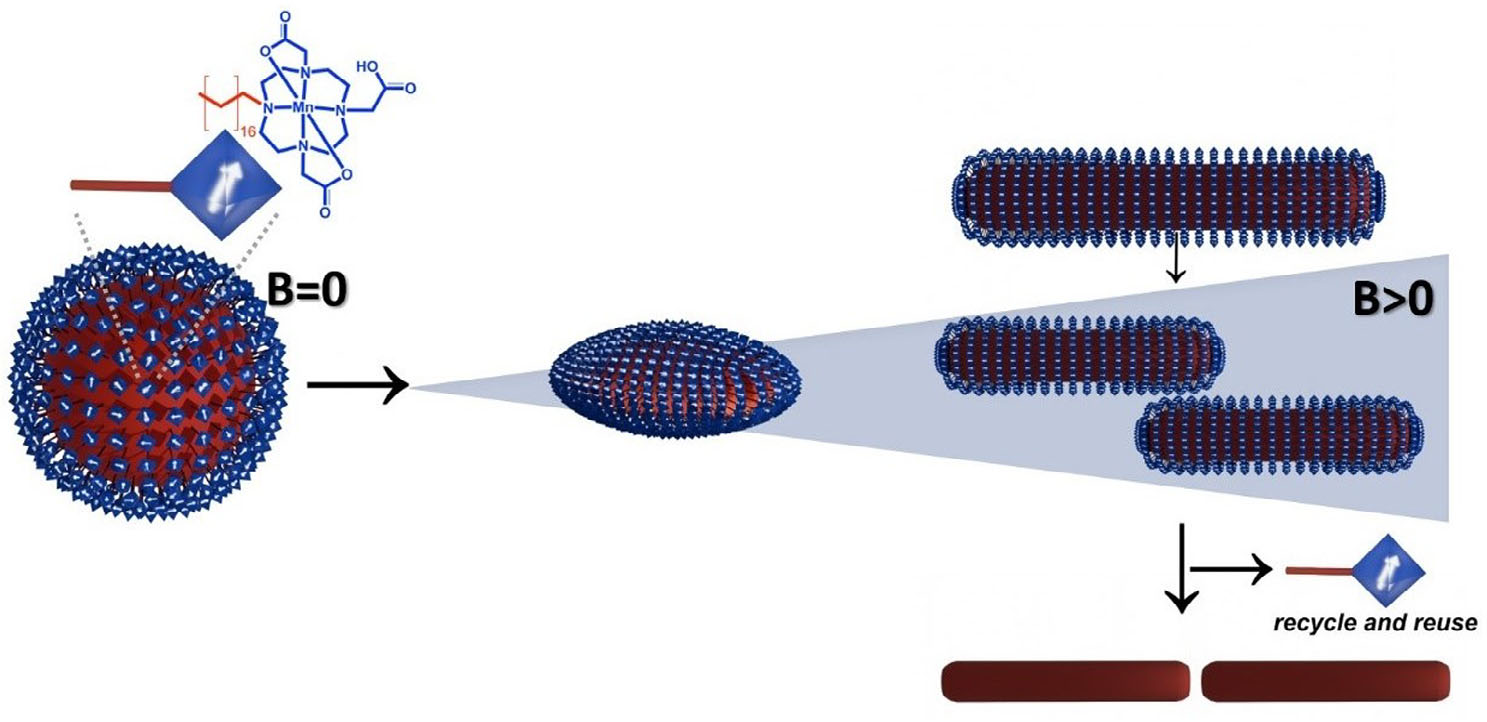
Proposed mechanism. A spherical emulsion droplet is stabilized by MagSurfs, which are also shown as Lewis structures (left). Because of the paramagnetic nature of the magnetic metal ion in the head group of the MagSurf, the orientation of the magnetic dipoles on the emulsion droplet is random. When field B is turned on, the magnetic dipoles orient in the field direction, exerting a torque on the surrounding molecules. The surfactants can no longer maintain the spherical shape and start to deform in the direction of the applied magnetic field and the emulsion droplets elongate (middle). As the strength of the applied field increases, the nanoparticle aspect ratio increases. Our data indicate a critical value for the elongation when the particles break up (right). Precipitation of the polymer delivers the final surfactant‐free nanorods (bottom right).

The results to this point indicate that the surfactant is an emulsifier only, and it does not become incorporated into the particles' polymeric matrix. We can propose a mechanism for the emergence of the shape anisotropy (Scheme 1). In the absence of a magnetic field, styrene's emulsification yields usual spherical droplets stabilized by MagSurf, which is paramagnetic, and therefore the Mn^2+^ magnetic dipoles are randomly oriented and rotate freely. The surfactant adapts to the droplet's curvature, which minimizes the surface‐to‐volume ratio. The situation changes in a magnetic field due to Mn^2+^ being bound to the octahedral ligand field of the DOTA head group. It is well documented in the literature that the DOTA‐ligand coordinating with metals creates a magnetic anisotropy.^[^ ^20^ ^]^ This anisotropy and the fact that Mn^2+^ is positioned in the head group create a torque to the molecule due to the magnetic polarization.

Because of the novelty of magnetic surfactants, there is no literature yet that gives a precise and full physical explanation for the observed phenomena. However, few papers address droplets filled with a dispersion of magnetic particles.^[^ ^21^ ^]^ Previous works have shown that magnetic fields facilitate the precise and contactless manipulation of MagSurf droplets, effectuating controlled deformations that overcome gravitational and interfacial forces. This phenomenon, driven by the principle of energy minimization, enables the active shaping. Predictable morphological transformations, such as shape alterations and potential swelling, are effectively captured by curvature elasticity models, thereby providing robust theoretical and experimental support for the viability of externally guided droplet manipulation.^[^ ^22^, ^23^, ^24^ ^]^

Calculations predict the deformation in an external magnetic field as a consequence of bending elasticity of the membrane and chaining through dipole–dipole interactions.^[^ ^25^ ^]^ Interestingly, the predicted shapes correspond very well to the banana‐like polystyrene particles we have observed (Figure 1b) but at a much lower field strength than assumed in the calculations (0.22 T instead of 10 T). The stronger the external B‐field gets, the higher the tendency of the surfactants to adopt a structure characterized by lower curvature. The emulsion droplet is deformed, flattens, and increasingly elongates (Scheme 1). Because the elongated emulsion droplets align in the magnetic field, one expects the emergence of an optical anisotropy (see Figure S10). Optical birefringence measurements show that the deformation process occurs within seconds after the magnetic field has been switched on and that the majority of the deformation process occurs at fields between 0 and 0.7 T. At even stronger fields, one would expect that the droplets become thinner and thinner as they elongate more and more. There has to be a limit. One reason is that the elastic deformation of the emulsion droplet increases the surface‐to‐volume ratio, while the number of available surfactant molecules is constant and thus limited. Another observation confirms the assumption that the deformation/thinning process could have a critical value. Figure 1f compares the volume of the latex particles produced at different field strengths. Notably, the volume of the elongated particles is roughly 7× times smaller than the spherical particles. The latter result can be explained by the emulsion droplets breaking apart into smaller nanoreactors in which the generation of the polymer particles takes place. The ^1^H‐NMR spectra of the dissolved polymer particles allow estimation of the average polymerization degree *P* by end group analysis and the resulting average molecular mass *M*_w_. The result is *P* ≈ 270 (*M*_w_ ≈ 28 000 g mol^−1^) for the spherical particles (*B* = 0 T). Considering a volume of the spherical particles of *V* ≈ 2.7 × 10^6^ nm^3^ (Figure 1f) together with the density of polystyrene (1.05 g cm^−3^), each particle consists of ≈1.65 × 10^7^ styrene units and ≈6 × 10^4^ polymer chains. Interestingly, the values differ for the PS nanorods (*B* = 9 T): *P* ≈ 160 (*M*_w_ ≈ 17 000 g mol^−1^). A nanorod is composed of ≈1.4 × 10^4^ polymer chains. Thus, there are roughly 4× times more polymer chains in the spherical particles compared to the elongated particles.

The semiquantitative calculations underline our assumption that the magnetically induced deformation leads to fragmentation at a critical value. Other reports suggest that critical deformation and fragmentation occurs when the neck diameter between two droplets becomes smaller than the radius of the smaller droplet, leading to breakage. A simplified criterion based on the neck‐to‐radius ratio is proposed. This process can have a significant impact on the size and morphology of nanoparticles, with particle size playing a crucial role in determining both stability and functionality.^[^ ^26^ ^]^ This explains why increasing the magnetic field shows a robust damping factor regarding the aspect ratio of the particles.

### Preparation of Polymer Nanorods from Arbitrary Polymers

The proposed mechanism builds on the presence of MagSurf at the emulsion droplet surface, functioning as a nanoreactor and enabling deformation under an external magnetic field. This concept is further reinforced by the introduction of two additional monomers, highlighting the versatility of our method. Compared to the preparation of polymer particles with micrometer length in templates such as anodically etched aluminum oxide membranes,^[^ ^27^, ^28^, ^29^ ^]^ our method simplifies the making of polymer‐based nanoparticles, which are metal free and with an adjustable aspect ratio between 1 and ≈6. Two different polymers have been selected to prove the broad applicability of the method: polymethylmethacrylate (PMMA) and polythiophene (PT), the latter representative of conducting polymers. PT nanorods are unknown as far as we can tell after an extensive literature survey.

**Figure 3:**
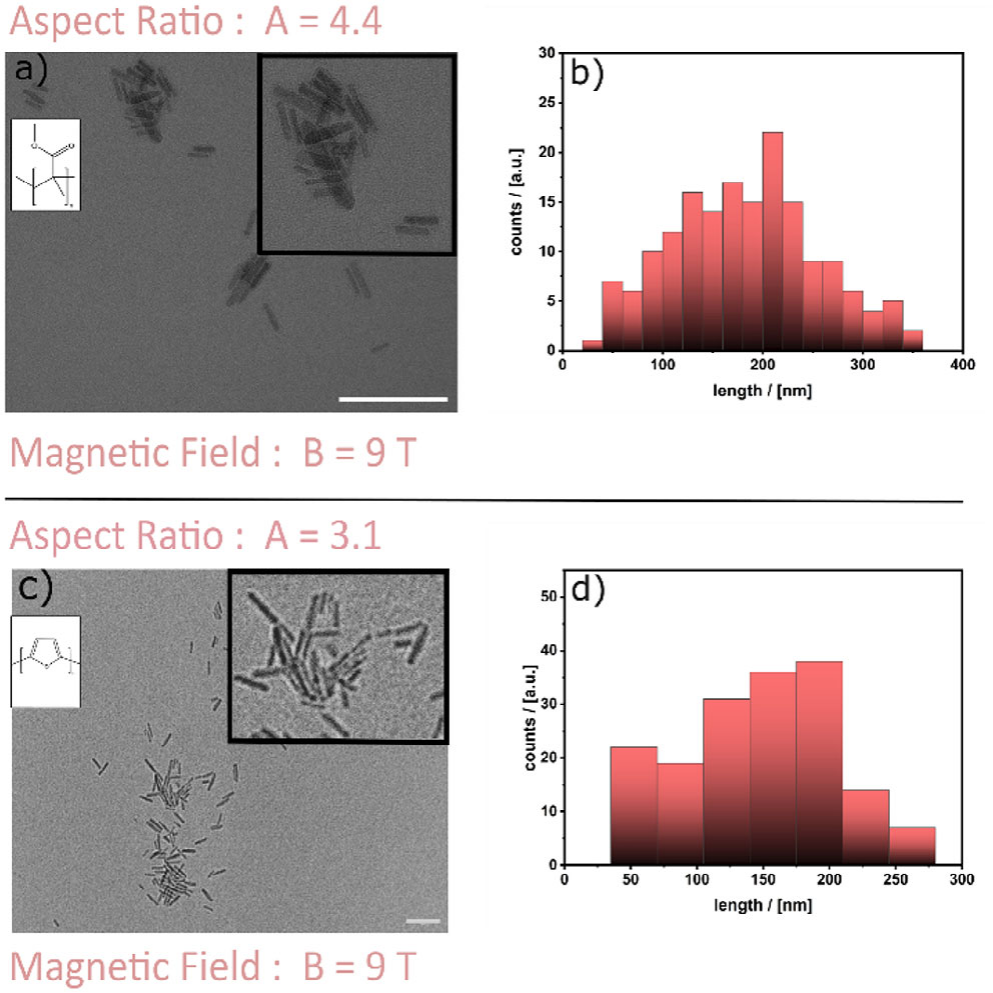
TEM micrographs (scalebar = 100 nm) of PMMA a) and PT c) prepared by emulsion polymerization using MagSurf as an emulsifier at *B* = 9T and the evaluation of the particle length distribution (b: *σ* = 10.016, d: *σ* = 44.147).

Both systems deliver the expected spherical polymer particles without a magnetic field (Figure S11). The results of performing the synthesis inside a magnetic field (*B* = 9 T) are shown in Figure 3. One can clearly see that polymer nanorods are obtained. Further data for the characterization of the samples are shown in the Figures. S12 and S13. Compared to the PS nanorods, the averaged aspect ratio is now lower (4.4 for PMMA; 3.1 for PT).

## Conclusion

This innovative technique harnesses the influence of magnetic fields on manganese‐complexed MagSurf during emulsion polymerization, resulting in notable alterations in particle morphology. When subjected to an applied magnetic field, nanoparticles transform from spherical to progressively curved shapes, ultimately evolving into rod‐like structures with aspect ratios reaching saturation at approximately 750 mT. With the creation of polythiophene nanorods, we have expanded the horizons for their application and facilitated the synthesis of previously rare PMMA nanorods. Our findings introduce a sustainable, streamlined method for crafting diverse anisotropic polymer‐based nanoparticles without post‐synthetic processing or harmful solvents. This approach enhances both efficiency and eco‐friendliness as MagSurf's reusability minimizes resource consumption and simplifies particle separation from the aqueous phase.

## Supporting Information

A detailed description og the experimental procedures and the data that support the finding of this study are available within the paper and its Supplementary Information. The authors have cited additional references within the Supporting Information.^[30–36]^

## Conflict of Interests

The authors declare no conflict of interest.

## Supporting information



Supporting Information

## Data Availability

The data that support the findings of this study are available in the supplementary material of this article.
