# Supplementary material for: A Versatile Route to Shape Polymer Nanoparticles by Deforming Nanoreactors Made from Magnetic Surfactants
Source: Angew Chem Int Ed Engl. 2025 Mar 26;64(21):e202422439. doi: 10.1002/anie.202422439 (PMC12087829; doi:10.1002/anie.202422439)
Supplement: Supplementary file 1 — Supporting Information [file ANIE-64-e202422439-s001.docx]

Supplementary Materials for

**A Versatile Route to Shaping Polymer Nanoparticles by Deforming Nanoreactors Made from Magnetic Surfactants**

Benjamin Botev, Stephan Siroky, Irene Morales, Sebastian Polarz^*^

Corresponding author: [sebastian.polarz@aca.uni-hannover.de](mailto:sebastian.polarz@aca.uni-hannover.de)

**The PDF file includes:**

Materials and Methods

Supplementary Text

Schemes S1 to S4

Figures S1 to S17

Table S1

Attachements

References

Materials and Methods

General Remarks

Unless otherwise specified, all reagents were purchased from commercial suppliers and used without further purification. Unless otherwise stated, all solvents were used as described in the experimental part. Nitrogen from Linde was used as the shielding gas for the syntheses, unless otherwise stated. All indicated yields refer to spectroscopically pure compounds, unless otherwise stated. The NMR spectra were recorded with a JEOL 400 MHz NMR spectrometer at 298 K in suitable deuterated solvents. The only exception is the introduction of DMSO, which was measured at 313.15 K. The chemical shifts of the protons are given in ppm (δ) relative to the resonance of the solvent resonance used as internal standard (CDCl_3_ δ 7.26 ppm; D_2_O δ 2.40 ppm). All spectra were recorded at 298 K unless otherwise noted and processed with MestreNova 14.2.3. Infrared-spectroscopy was performed on an Alpha II ATR IR from Bruker. An Amazon Speed Etd mass spectrometer from Bruker was used to measure the mass spectra. EPR spectra were recorded on a table-top X-band spectrometer MiniScope MS 500 (Bruker-Magnet-Tech) equipped with a temperature controller H03 (Bruker-Magnet-Tech). The Zetasizer pro from Malvern was used to measure the surfactant aggregates and emulsion droplets. The emulsions were dispersed with an ultrasonic bath from Sonorex. A CX41 polarization microscope from Olympus was used for the polarization microscopic images. The TEM images were taken with the HT7900 device from Hitachi and Olympus camera. The EDX measurements were carried out on a Regulus 8230 from Hitachi using an Oxford 100 mm^2^ windowless EDX-Detector. The plotting of all spectra was carried out with OriginLab 2023b. The figures to illustrate the mechanistic conditions were created with Inkscape and Blender. Fiji was used to analyze particles and determine their distribution. All glassware, stirring rods and caps used for the synthesis were dried in an oven at 60 °C for at least twelve hours. The reactions were carried out in the absence of air and moisture. The chemicals used were added to the reaction flasks via septa using syringes and a stream of nitrogen. The reactions at μ_0_H = 0.75 T were done by a Hallbach rebuild setup. For the achievement of the magnetic field of μ_0_H = 9 T we placed a sample-tube with the reaction mixture into the NMR for 45 min. The magnetic field of 0,22 T was created by an own build application. The monomers styrene and methyl methacrylate were used to produce the nanoparticles.

The monomers were dried over calcium hydride, stored at -18° C and freshly distilled before each preparation. For the Formation of emulsions, Surfactants MnC_18_DOTA were dissolved in water (Mili-Q-standard) resulting in clear solutions. Micro emulsions were prepared by ultrasonication treatment within 300 sec. followed by shaking for a duration of 120 sec resulting in clear solutions. The resulting micro emulsions were analyzed by dynamic light scattering.

| **Chemical** | **Supplyer** | **CAS** |
| --- | --- | --- |
| Cyclene | abcr | 294-90-6 |
| *Tert-*butyl-bromoacetate | TCI | 5292-43-4 |
| Chloroform | Roth | 67-66-3 |
| Triethylamine | Sigma-Aldrich | 121-44-8 |
| Magnesiumsulfate | Applichem | 7487-88-9 |
| *N,N*-dimethylacetamide | Sigma-Aldrich | 127-19-5 |
| Potassium hydrogen carbonate | Applichem | 144-55-8 |
| Diethylether | Roth | 60-29-7 |
| *1-*Bromooctodecane | Sigma Aldrich | 112-89-0 |
| Trifluoroacetic acid | abcr | 76-05-1 |
| Methanol | Roth | 67-56-1 |
| Manganese-(II) carbonate | Applichem | 546-93-0 |
| Dichlormethane | Roth | 75-09-2 |
| Styrene | Merck | 100-42-5 |
| Methyl methacrylate | Sigma-Aldrich | 80-62-6 |
| Thiophen | TCI | 110-02-1 |

Scheme S1


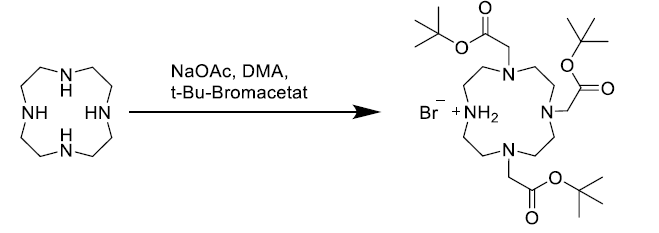


| (**1**) | (**2**) |
| --- | --- |

As first step, commercially available cyclen (**1**) was selectively tris-alkylated with *tert-*butyl bromoacetate to yield the hydrobromide salt of 1,4,7-*tris*(*tert*-butoxycarbonylmethyl)-1,4,7,10-tetraazacyclododecane (DOTA·HBr (**2**)). In a second step, the corresponding free base was afforded in excellent yields. The synthesis method of Jagadish et al ^[30]^ was reproduced leading to high yields of the tris-alkylated compound (**2**) owing to kinetic control during the nucleophilic substitution reaction at low temperatures. In brief, 5.00 g cyclen (29 mmol,1.0 eq.) and 7.87 g sodium acetate (96 mmol, 3.3 eq.) were dissolved in 60 mL *N,N*-dimethylacetamide (DMA) under vigorous stirring. 18.7 g *tert-*butyl bromoacetate (14.1 mL, 96 mmol, 3.3 eq.) in 20 mL DMA were added dropwise to the suspension at -20 °C. After vigorous stirring for 24 h, the reaction mixture was poured into 300 mL deionized water and 15 g KHCO_3_ were added. The precipitate was collected by filtration, redissolved in 250 mL CHCl_3_, washed with 100 mL deionized water, dried (MgSO_4_) and concentrated to 25 mL. For the precipitation of (**2**), 250 mL Et_2_O were added. Thus, the desired target product was precipitated accordingly and the di- and tetra-alkylated by-products can be separated in solution. After filtration, washing with Et_2_O and drying *in vacuo*, 5.00 g of (**2**) (8.4 mmol, 1.0 eq.)
Yield: 91%
^1^H NMR (400 MHz, CDCl_3_) δ 3.38 (s, 4H), 3.29 (s, 2H), 3.10 (s, 4H), 3.01 – 2.83 (m, 12H), 1.48 – 1.41 (m, 27H). ^13^C NMR (101 MHz, CHLOROFORM-*D*) δ 170.62, 169.73, 81.86 (d, *J* = 15.1 Hz), 77.32, 58.31, 51.43, 49.28, 47.61, 28.30 (d, *J* = 3.9 Hz).
*m/z* [M+H] calcd for C_26_H_51_N_4_O_6_Br: 588,38 found: 588,77

Scheme S2

| (**2**) | (**3**) |
| --- | --- |

0.50 g DO3A-t Bu (**2**) (1.00 mmol; 1.0 eq.) were dissolved in 20 mL CHCl_3_ under nitrogen gas atmosphere. 0.12 g triethylamine (1.20 mmol; 1.2 eq.) were added dropwise under stirring. The solution was heated to reflux and 0.33 g 1-Bromooctadecane (1.07 mmol; 1.1 eq.) dissolved in 10 mL CHCl_3_ were added dropwise. The reaction mixture was refluxed for further 24 h (N_2_). The organic phase was extracted with 20 mL distilled water to remove triethylamine salts and then dried (MgSO_4_). The solvent was removed by rotatory-evaporator and the obtained pale-yellow solid (**3**) was finally dried in vacuo.
Yield: 78%

^1^H NMR (400 MHz, CHLOROFORM-*D*) δ 3.41 – 2.21 (m, 26H), 1.47 – 1.34 (m, 29H), 1.33 – 1.14 (m, 32H), 0.89 – 0.80 (m, 3H). ^13^C NMR (101 MHz, CHLOROFORM-*D*) δ 77.32, 29.89 – 29.52 (m), 28.42 – 27.68
(m). *m/z* [M+H] calcd for C_45_H_88_N_4_O_6_: 780,67 found: 780,30

**Scheme S3**

| (**3**) | (**4**) |
| --- | --- |

In a typical literature-based TFA-deprotection procedure ^[31]^, 0.63 g DOTA-*t*-Bu-C18 (**3**) (0.85 mmol) were dissolved in 10 mL DCM and 20 mL TFA were added at 30 °C under nitrogen gas atmosphere. The reaction mixture was stirred for 24h. A brown-orange solution was obtained. The solvents were removed under reduced pressure. 1.0 g of a brown dark oil was afforded, which was purified via precipitation. The yellow oil was dissolved in 2 mL MeOH and 50 mL Et2O were added slowly at 0 °C. A yellow-brownish precipitate formed and the reaction mixture was stirred for further 4 h at 25 °C. The solvents were decanted and the remaining solid residue was washed several times with Et2O. C_18_DOTA-TFA (**4**) was afforded after a drying step in vacuo as white slightly yellowish solid.
Yield: 71%
^1^H NMR (400 MHz, D_2_O) δ 3.40 – 2.91 (m, 25H), 1.27 – 0.99 (m, 34H), 0.72 (d, *J* = 7.8 Hz, 3H). ^13^C NMR (101 MHz, DMSO-*D*_6_) δ 40.65, 40.29, 29.56 (d, *J* = 4.4 Hz).
*m/z* [M+H] calcd for C_32_H_62_N_4_O_6_: 612,47 found: 612

Scheme S4

| (**4**) | (**5**) |
| --- | --- |

200 mg (0.33 mmol, 1 eq.) of (**4**) and 44 mg (0.33 mmol) MnCO3∙H2O are solved in 6 mL Milli-Q water and stirred at 35 °C for 2 days. After cooling to room temperature, the reaction mixture was filtered with a syringe filter (VWR 0.45 μm Nylon) and lyophilized. MnC18Dota (**5**) was obtained as slightly yellow crystals in 100% yield (178 mg, 0.33 mmol).

*m/z* [M+H] calcd for C_32_H_60_N_4_O_6_Mn: 651,39 found: 649,79

**Additional analytics for MnC_18_DOTA**

Fig. S1


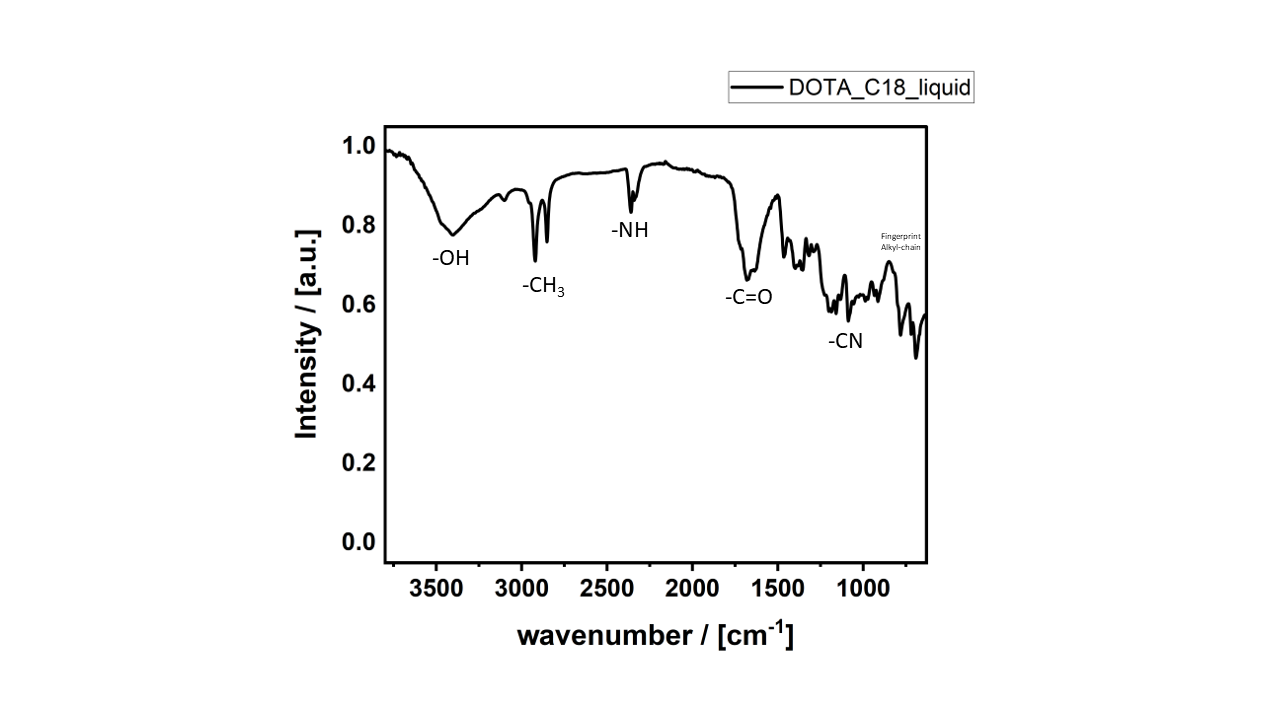


**Fig. S1:** IR Spectrum of the Mn_C_18__DOTA. the -OH band appears at approximately 3500 cm⁻¹. The neighboring absorption peak with two intensities can indeed be attributed to the symmetric and asymmetric motion of the terminal CH₃ group of the alkyl chain. The expected peak for the carboxylic acid is observed around 1600 cm⁻¹, while the region at 1200 cm⁻¹ reflects the nitrogen-carbon bonds of the cyclen-based headgroup. Everything beyond this area falls within the fingerprint region, which is defined by the C_18_ alkyl chain.

Fig. S2


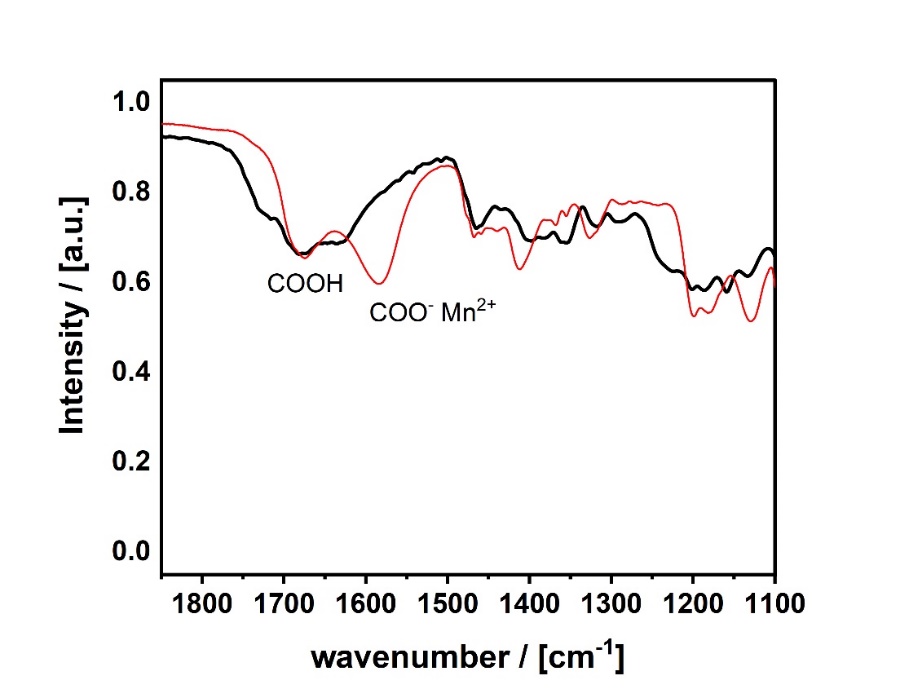


**Fig. S2:** Layered spectrums of the C_18__DOTA Ligand (black) and the C_18_DOTA_Mn (red). Regarding the characteristic vibrational modes of -COOH group, corresponding absorption bands appear in 1500 cm⁻¹ to 1600 cm⁻¹. Observing two distinct peaks in the red spectrum, which corresponds to the DOTA_C18_Mn complex. The lower-energy peak within this band can be attributed to the stretching vibration of the C=O double bond, whereas the higher-energy peak is associated with the free -OH group of the carboxyl moiety. ^[32]^ Two oxygen atoms from the carboxyl functional group interact with the metal center. One -OH group remains uncoordinated. Furthermore, the absence of a -COO⁻ group in the native DOTA ligand corroborates the emergence of the new spectral feature. Striking observing range at approx. 1200 cm^-1^, as the rigidity of the nitrogen atoms inhibits the vibrations due to the direct coordination with the metal on the -CN groups, affecting the mobility of the groups.

Fig. S3


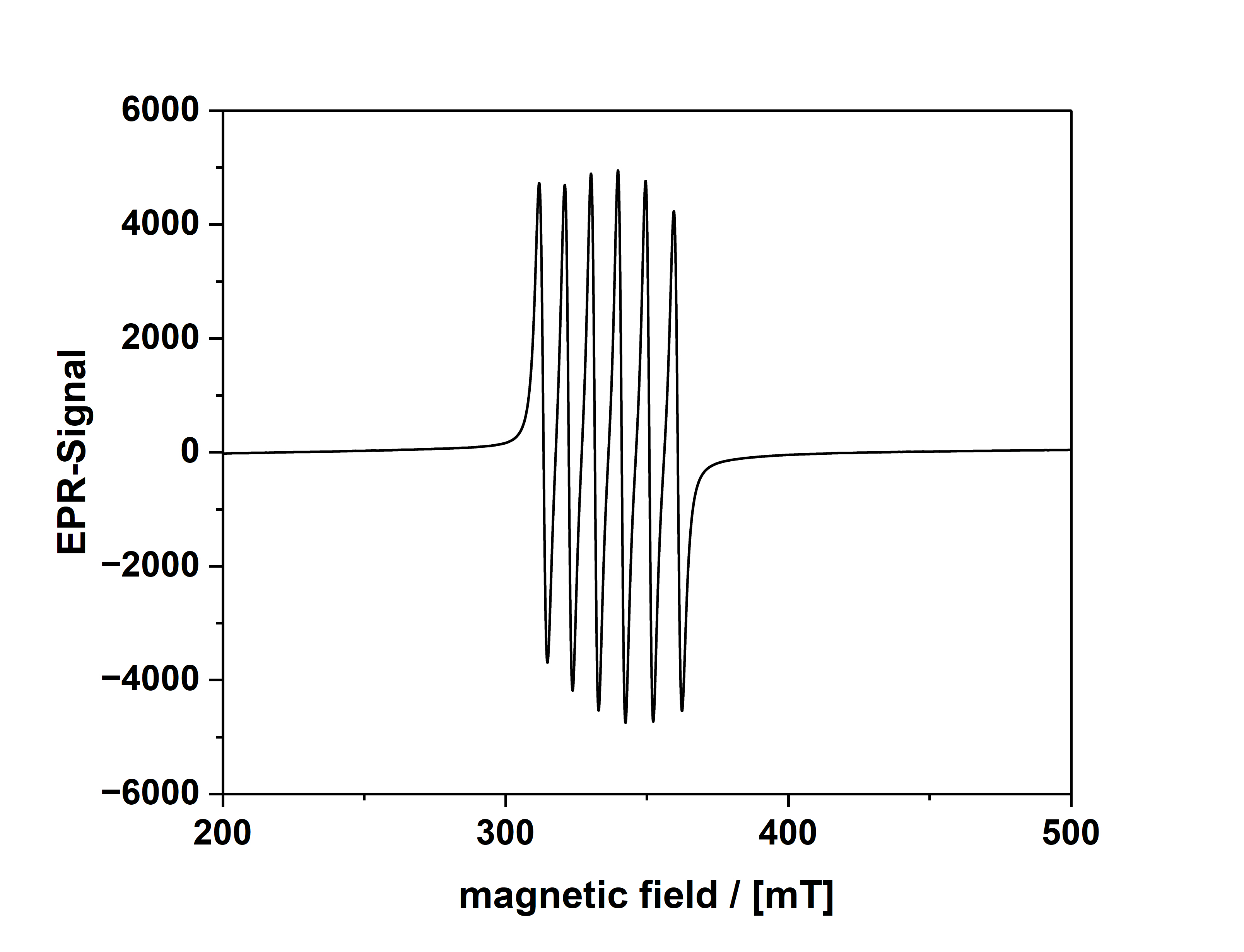


Fig. S3: EPR-spectrum of C_18__DOTA_Mn measured in aqueous solution and by 298.15 K.

Fig. S4

a)


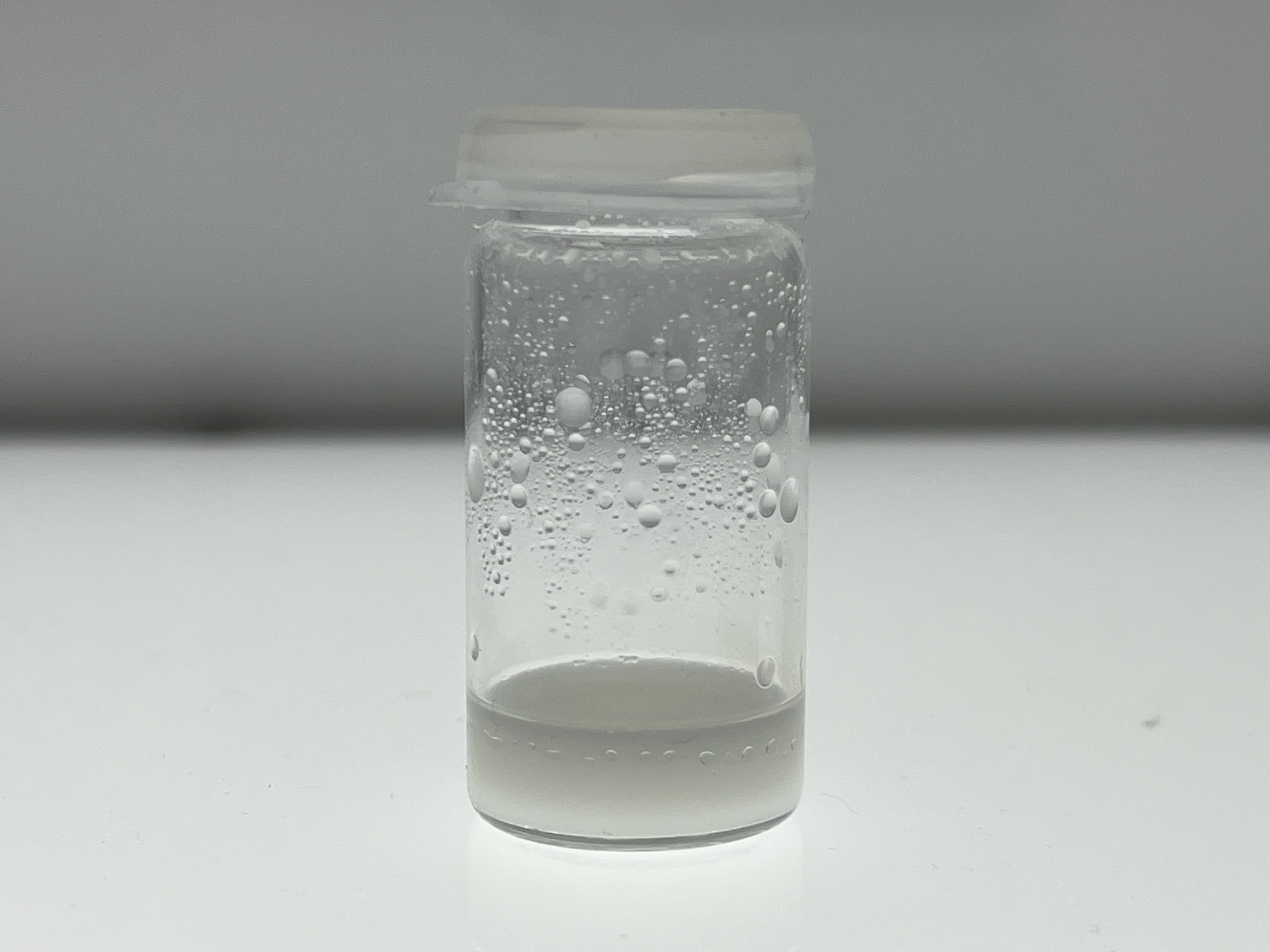


b)





Fig. S4: Photograph (a) and DLS (b) of an oil-in-water emulsion (20% styrene: 80% H_2_O) prepared using the MagSurf C_18__DOTA_Mn.

Fig. S5





(a): Particle size distribution obtained from DLS measurements of latex particles prepared under field-free conditions.


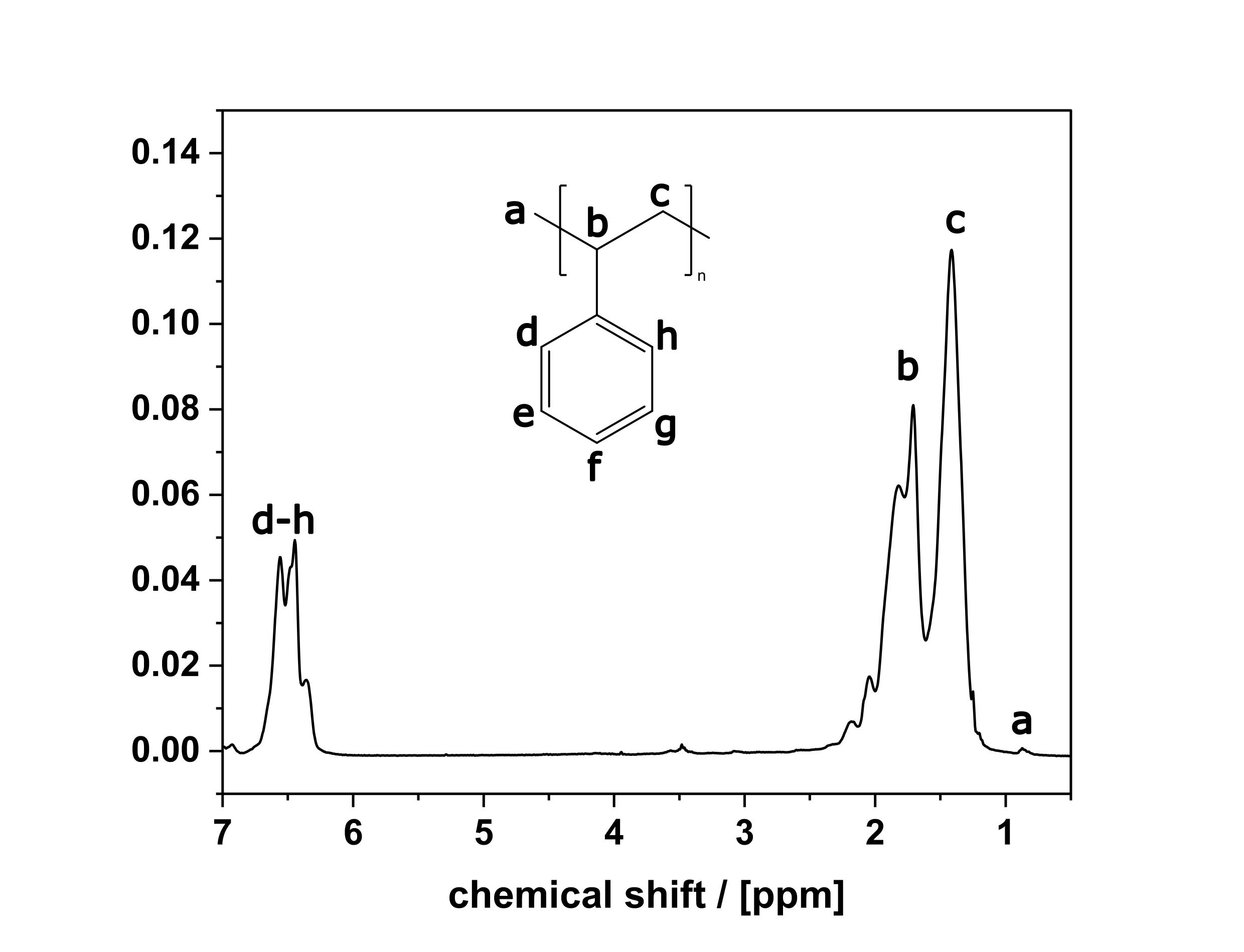


^1^H NMR (400 MHz, CHLOROFORM-*D*) δ 6.46 (dd, *J* = 52.0, 34.5 Hz, 343H), 2.19 (s, 692H), 1.45 (d, *J* = 34.1 Hz, 701H).

(b): ^1^H-NMR of polystyrene dissolved in CDCl3 (TMS). The polymer was synthesized from an oil-in-water emulsion stabilized by MagSurf, without a magnetic field. Aromatic protons are observed around 6 ppm. The alkyl chain is identified near 3 ppm. The approximate molar mass distribution was subsequently determined from the integrals obtained via end group analysis. The calculation follows a specification from the literature (3) the NMR spectra with the integrals are provided in the appendix. The values obtained are as follow on Page 13.

Table S1

Protons and integrals for end group analysis

| Protons | Integral |
| --- | --- |
| 7.13 ppm – 6.50 ppm | 342.71 |
| 1.52 ppm | 700.75 |
| 2.00 ppm | 691.98 |

Total of aromatic protons

342.71

Five aromatic protons per styrene unit:

$\frac{\boldsymbol{342.71}}{\boldsymbol{5}}$ = 68.54

Total of aliphatic protons:

700.75 + 691.98 = 1392.73

Three aliphatic protons per styrene unit:

$\frac{\boldsymbol{1392.72}}{\boldsymbol{3}}$ = 464.24

Due to different numbers of styrene units, calculating an average degree of polymerization:

$$\boldsymbol{n=}\frac{\mathbf{464.24+68.54}}{\mathbf{2}}\mathbf{=266.399}$$

Molar mass of a styrene unit is approximately 104.15 g/mol:

*M_w_* = 266.39 * 104.15 = 27704.73 g/mol

Fig. S6

Characterization of PS Nanorods prepared by emulsion polymerization using MagSurf as an emulsifier at B = 9T.

(a)





(a): Number values by DLS measurement of the PMMA based nanoparticles.

(b)





(b): Distribution of the Aspect Ratio of the Polystyrene nanorods.

(c)





^1^H NMR (400 MHz, CHLOROFORM-*D*) δ 7.05 (d, *J* = 25.8 Hz, 448H), 6.51 (d, *J* = 50.3 Hz, 279H), 1.83 (s, 224H), 1.41 (s, 300H).

The compound was measured with a solvent suppressing program, which therefore only shows the polymer peaks, the program is part of the JEOL NMR and is called 1D WET. The molar mass distribution was also determined using 1H NMR as in Fig. S9. A figure of the NMR spectrum including integrals can be found in the appendix. The results were as follows.

Average degree of polymerization: *n* = 160.04

Estimated molar mass distribution: *M_n_* = 16644.17 g/mol

Fig. S7


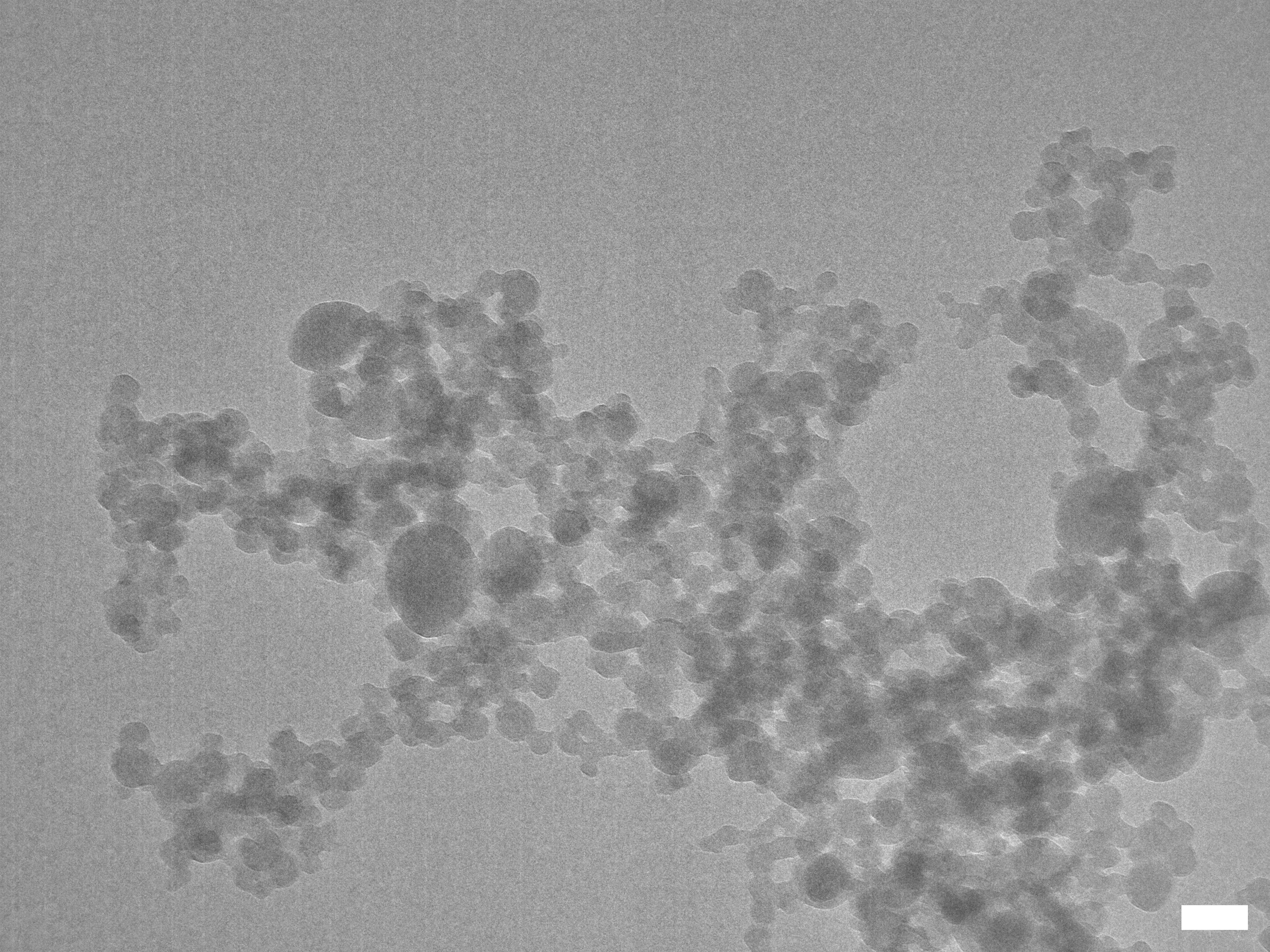


(a): TEM micrograph of PS-particles prepared using C_18_DOTA in B = 9T.





(b): Diameter distribution of the spherical polystyrene nanoparticles by using C_18_DOTA Ligand surfactant as emulsifier.

Fig. S8

Magnet setups


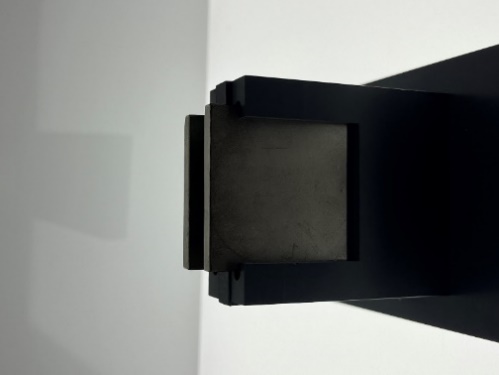
a)


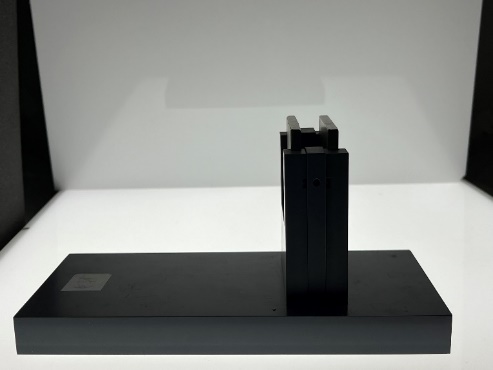


b)


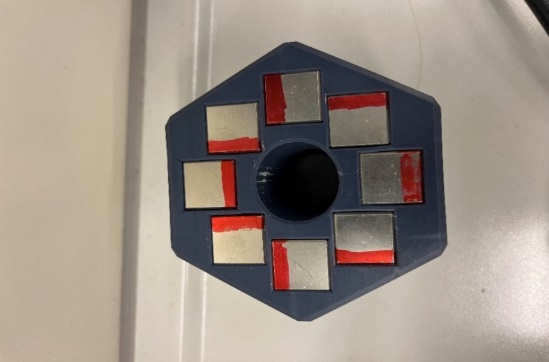

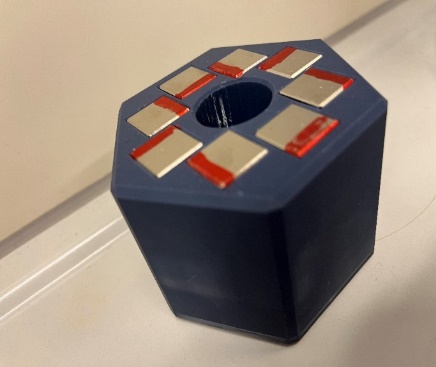


Figure (a) illustrates a setup comprising two magnetic plates positioned at a minimal separation, allowing for the insertion of a cuvette between them; the homogeneous magnetic field within this configuration measures 0.22 T. Figure (b) depicts a setup designed in accordance with a Halbach array and follows a specification detailed in the literature. The homogeneous magnetic field within this configuration measures 0.75 T. ^[33]^

Fig. S9

Analysis of the supernatant after PS polymerization.


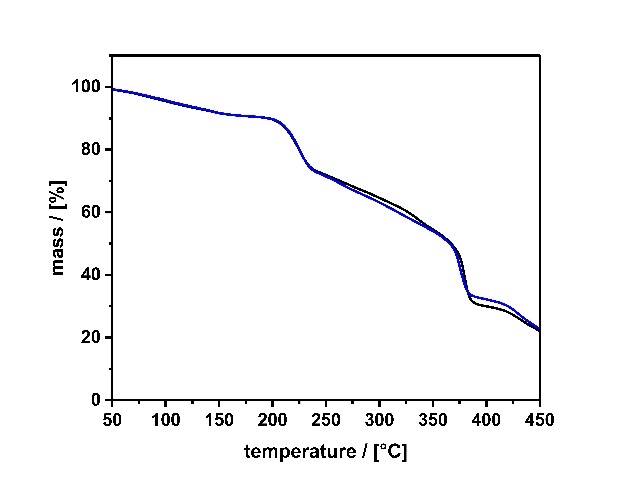


(a) TGA traces recorded in air (20% O_2_) of the as-prepared C_18__DOTA_Mn (black) and the compound isolated (blue) from the supernatant after polymer synthesis.





(b) IR-Spectrum of C_18__DOTA_Mn (black) and the recovered (red) sample.


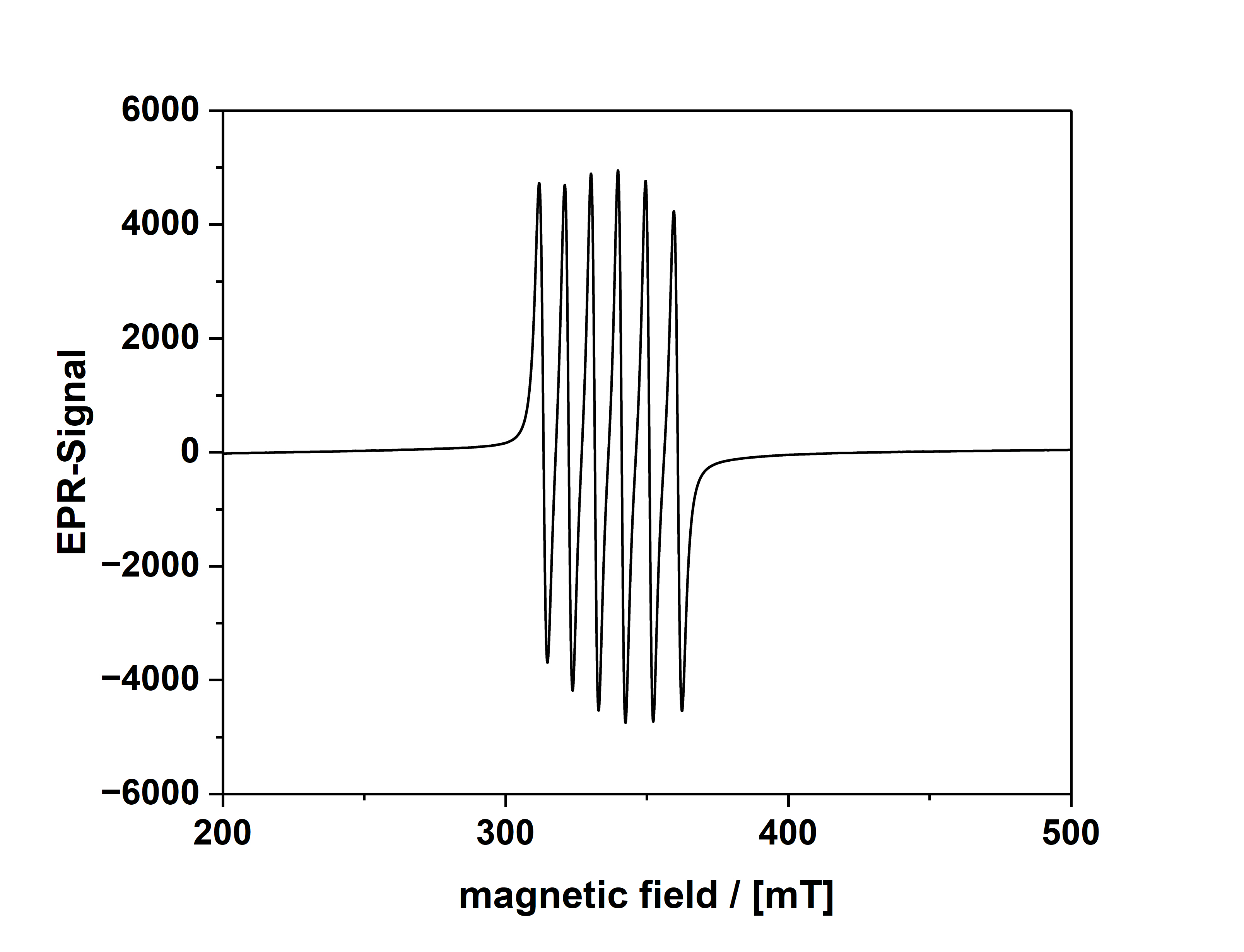


(c) EPR spectrum of the compound isolated from the supernatant after polymer synthesis, showing the typical sixfold peak for a Mn^2+^ species.

Detection of the absence of manganese after processing.

(d) ICP-OES analysis of the centrifuged polymer material, following the washing of the suspension, indicates that manganese is not detectable within the measurement limit. Only a trace amount, below the detection threshold, was observed. After washing, 2 mg of the polystyrene mass was analyzed. The measurement evaluation revealed a concentration of 0.006 mg/l, which is below the detection limit





(e) EPR spectrum of the final washed and centrifuged suspension, it shows no peak signal for Mn^2+^.





(f) The data recorded in the SEM-EDX show that there is primarily a high amount of carbon on the selected area, which is common for polystyrene, as well as a high amount of Silicium, Aluminium and Copper, which are the material that builds the sample-carrier.

Fig. S10


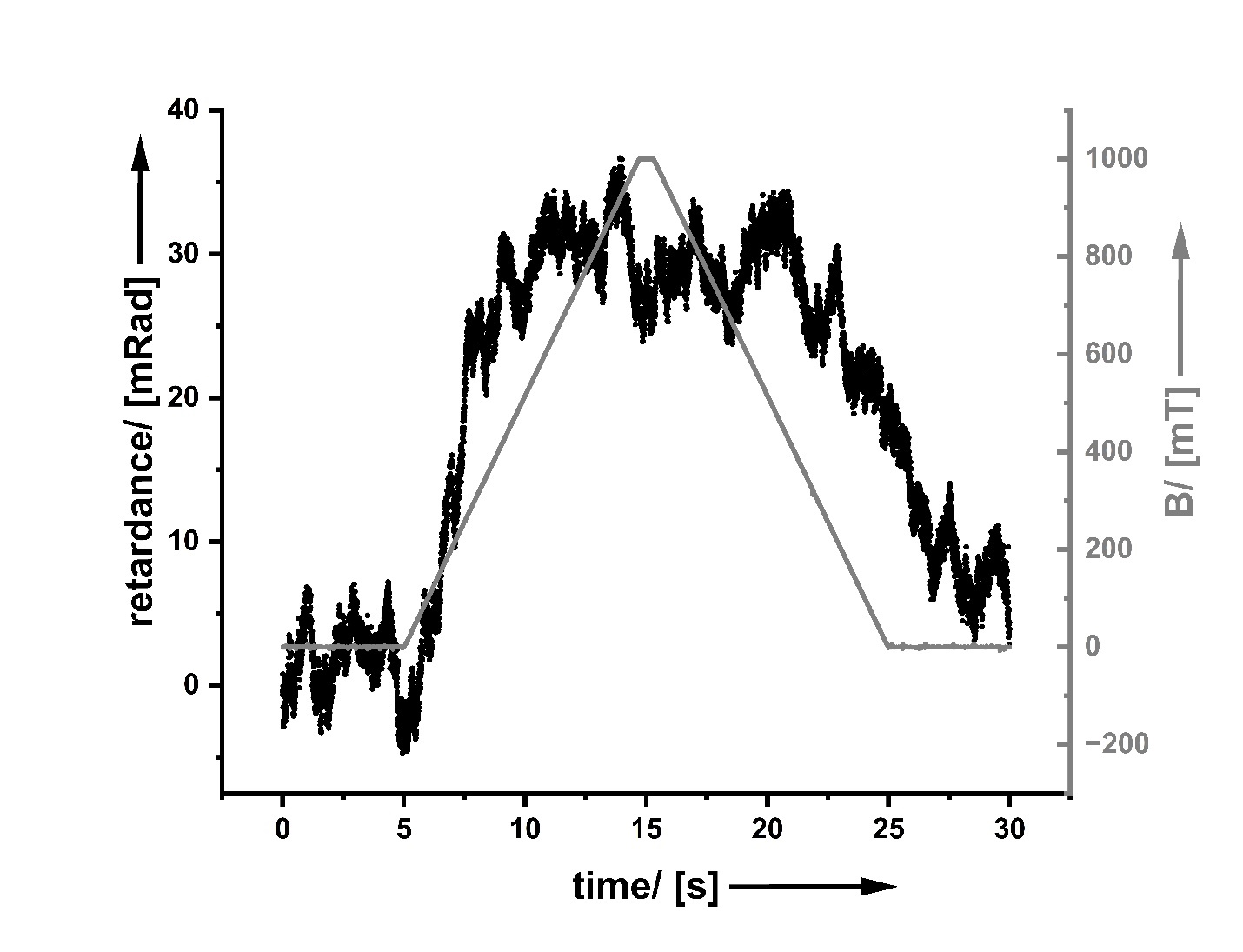


**Figure S10:** Optical birefringence measurements of an oil-in-water emulsion stabilized by a magnetic DOTA-based surfactant. The grey line shows the strength of the applied magnetic field as a function of time. The black data points show the birefringence. The sample is optically isotropic in the beginning as expected for spherical emulsion droplets. As soon as the magnetic field is turned on, one sees a birefringence signal. This can be explained by the deformation and alignment of the droplets in the magnetic field. Similar to the findings described in the main text of the paper, the major deformation process occurs for a field between 0-0.7 T. Stronger fields have only a minor effect.

Fig. S11

(a) (b)


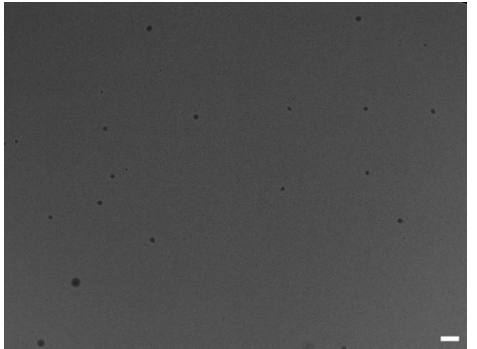

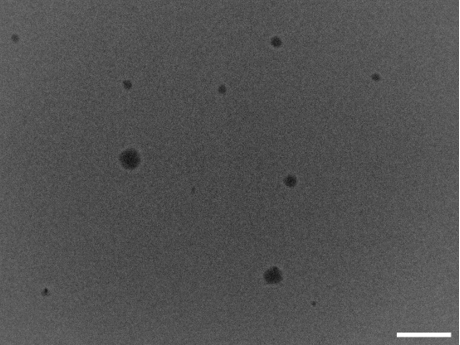


Fig. S11: Tem micrographs of PMMA (a: scalebar = 100 nm) and PT (b: scale bar = 100 nm) prepared by emulsion polymerization using MagSurf as an emulsifier at B = 0T.

Fig S12

Characterization of PMMA prepared by emulsion polymerization using MagSurf as an emulsifier at B = 9T.





^1^H NMR (400 MHz, CHLOROFORM-*D*) δ 3.61 (d, *J* = 19.6 Hz, 313H), 1.85 (d, *J* = 34.4 Hz, 139H), 0.99 (d, *J* = 18.5 Hz, 138H), 0.86 (d, *J* = 17.3 Hz, 150H).

(a): The peaks are broad, as is typical for polymer materials. In (a), the terminal CH_3_ group at the linkage is shown. In (b), the CH_2_ units along the chain are observed. Finally, (c) displays the free CH3 group at the oxygen, showing the characteristic –OCH_3_ shift. The approximate molar mass distribution was also determined by ^1^H NMR spectroscopy and end group analysis.

Average degree of polymerization: *n* = 247.50

Estimated molar mass distribution: *M_n_* = 24780.03 g/mol





(b): Number values by DLS measurement of the PMMA based nanoparticles.





(c): Distribution of the lengths of the PMMA nanoparticles.


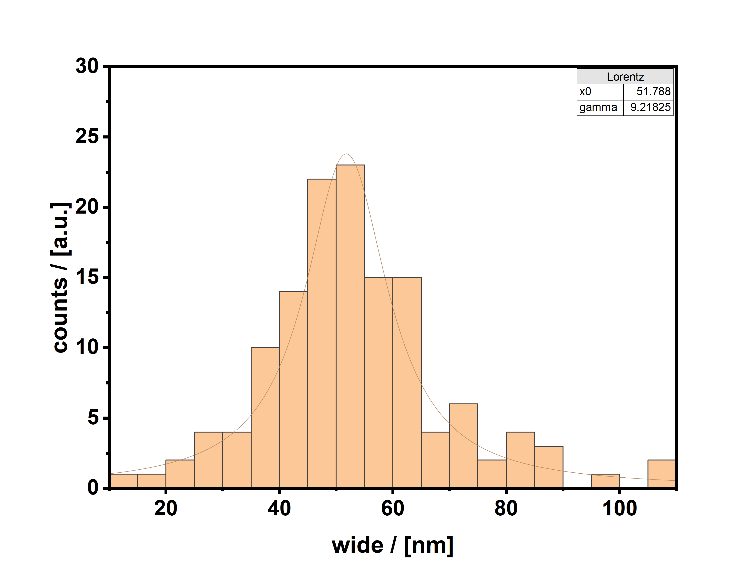


(d): Distribution of the width of the PMMA nanoparticles.





(e): Distribution of the Aspect Ratio of the PMMA nanoparticles.

Fig S13

Characterization of PT prepared by emulsion polymerization using MagSurf as an emulsifier at B = 9T.





^1^H NMR (400 MHz, CHLOROFORM-*D*) δ 5.90 (s, 52H), 3.61 (s, 146H), 3.02 (s, 3H).

(a):^1^H-NMR spectrum of PT-based nanoparticles. The peaks are shifted to the right by approximately 1 ppm, with a broadening of about 5 ppm. Unusual chemical shifts are observed for protons on the thiophene rings, such as shifts to lower values due to π-stacking. A signal at 6 ppm indicates unusual shielding from the π-system of an adjacent thiophene ring. This π-stacking is plausible in polythiophenes due to their entanglement, causing alkyl chains to lie close to the π-systems, which may deshield the alkyl protons and result in shifts to higher ppm values ^[34,35]^. The approximate molar mass distribution was also determined by ^1^H-NMR spectroscopy and end group analysis.

Average degree of polymerization: *n* = 148.87

Estimated molar mass distribution: *M_n_* = 12525.93 g/mol





(b): DLS Spectrum of the PT based nanoparticles.





(c): Distribution of the lengths of the PT nanoparticles.





(d): Distribution of the width of the PT nanoparticles.





(f): Distribution of the Aspect Ratio of the PT nanoparticles.

NMR Attachements

DOTA-HBr








DOTA_C18 (Protecting Groups)


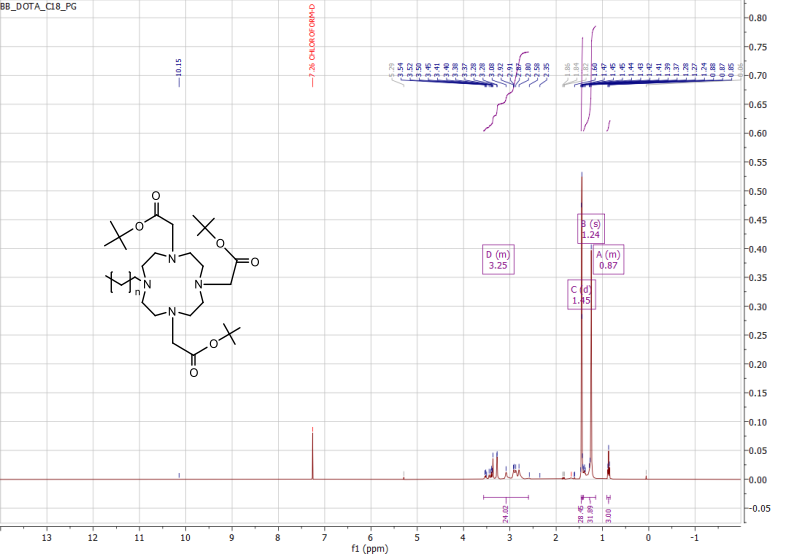





DOTA_C18_Ligand








Polymer nanoparticles were also analyzed by ^1^H-NMR, from samples with and without a magnetic field during the reaction to confirm the polymer species and estimate the molar mass distribution via endgroup analysis.

**Polystyrene Spherical shaped particles (without an external magnetic field)**

**
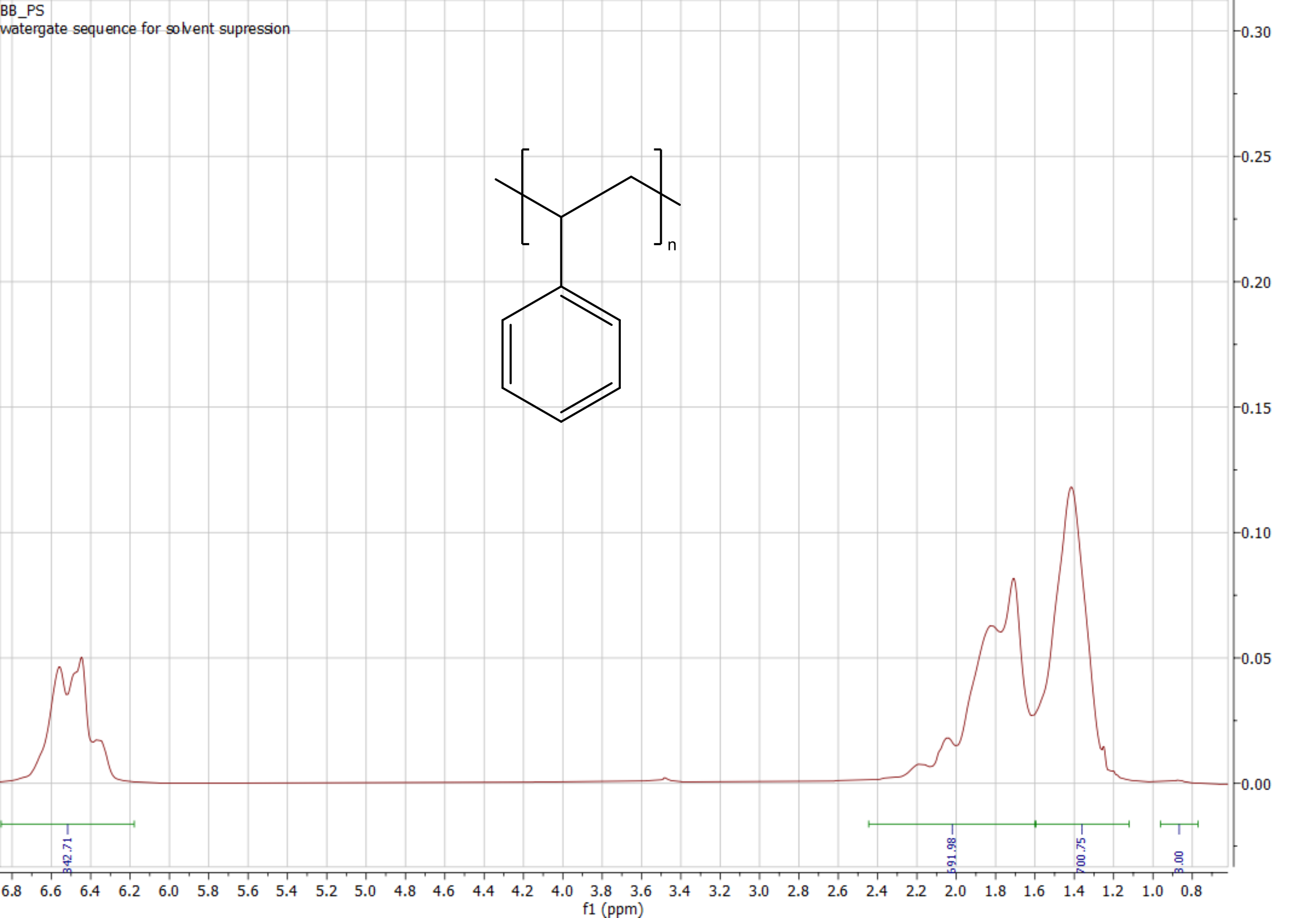
**

**Polystyrene Rod-shaped particles (reaction with an external magnetic field)**

**
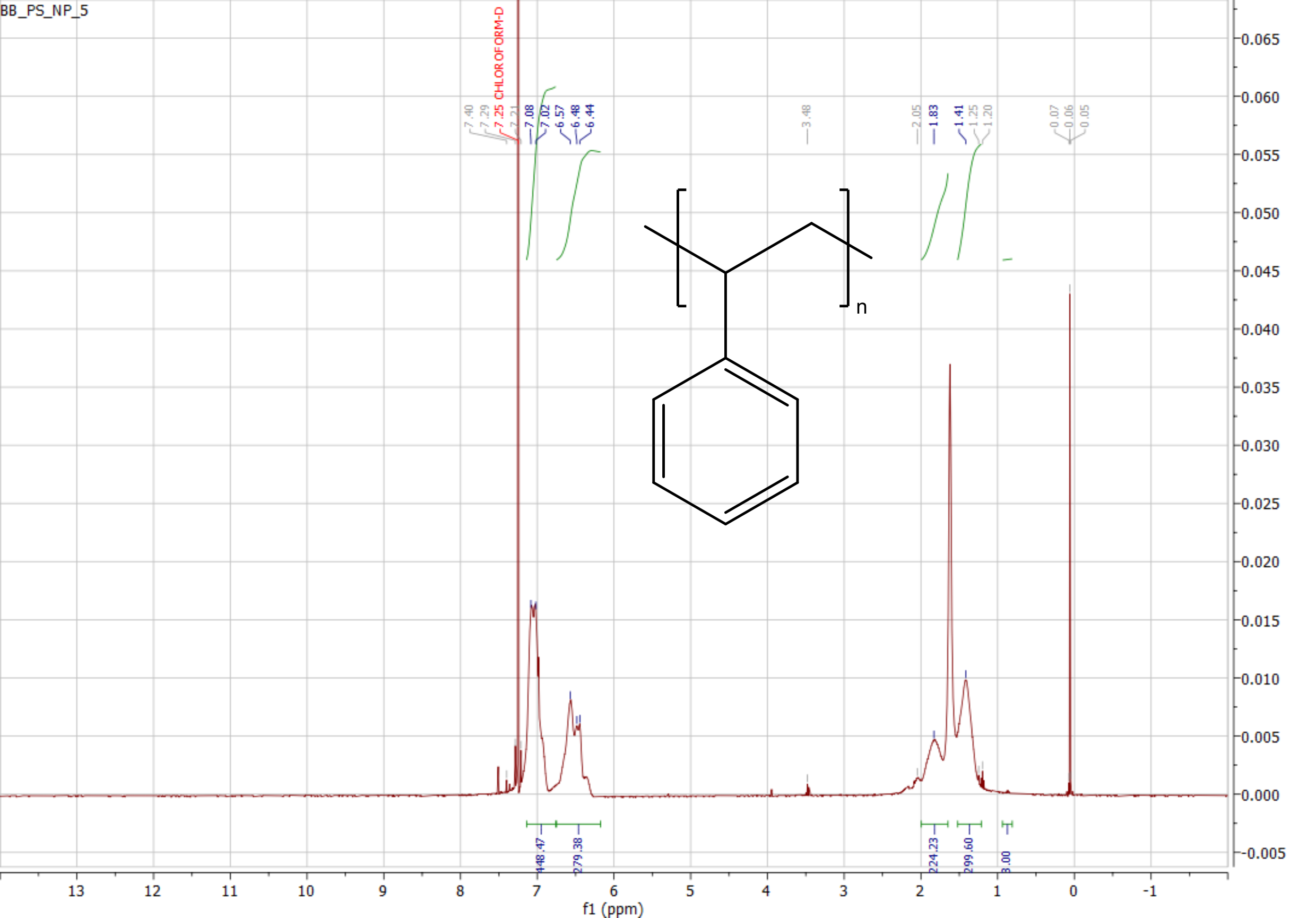
**

**PMMA Rod-shaped particles**

**
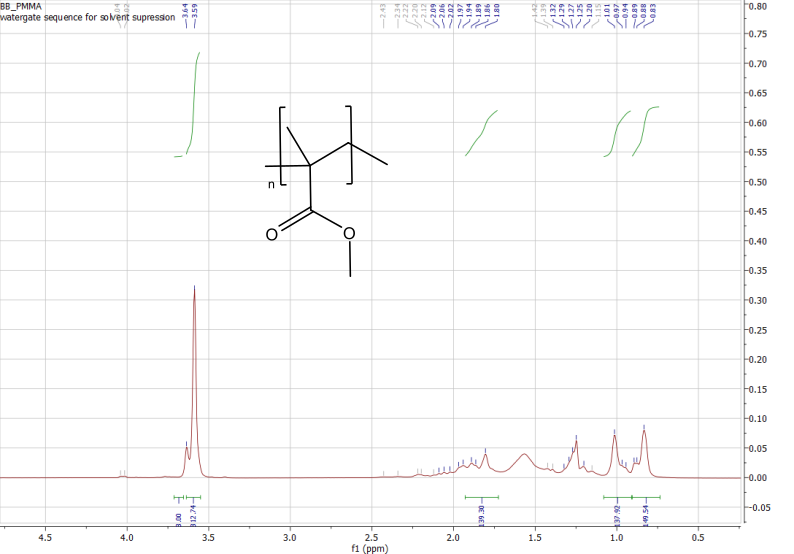
**

**PT Rod-shaped particles (1D WET measure setup)**

**

**

**EDX Attachement**

**Polystyrene nanorods (from a reaction at B_0_ = 9T)**

**
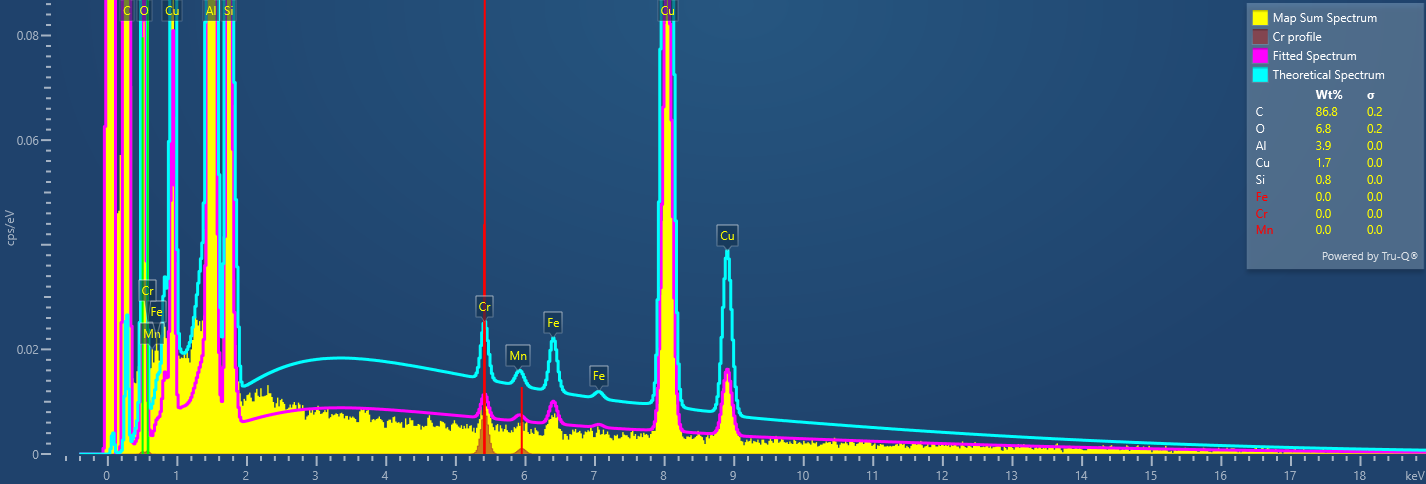
**

**
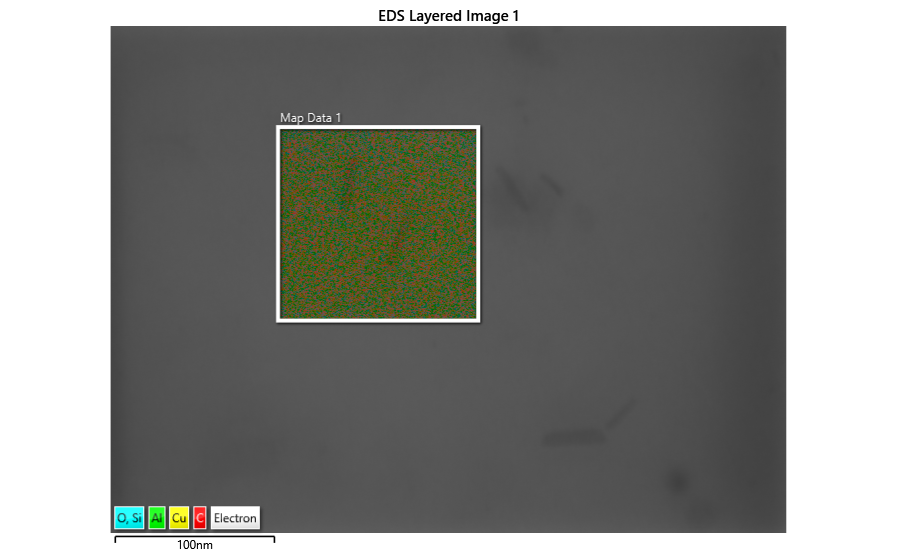
**

**Structure Attachement**

**DOTA headgroup complexed with Mn^2+^ (Solid State).**

Manganese (II) prefers sixfold coordination due to its d⁵-electron configuration, stabilizing an octahedral geometry. DOTA donates four nitrogens, but steric limits restrict Mn (II) to coordinating four nitrogens and two carboxylates, leaving one free group for hydrophilicity. In contrast, larger dysprosium (III) favors sevenfold coordination by binding an extra carboxylate. This highlights DOTA’s adaptability to metal ion size and preferences.

**
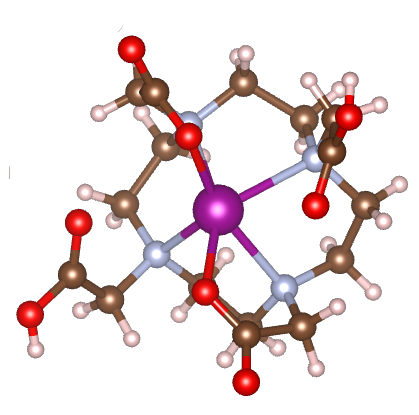
**

**
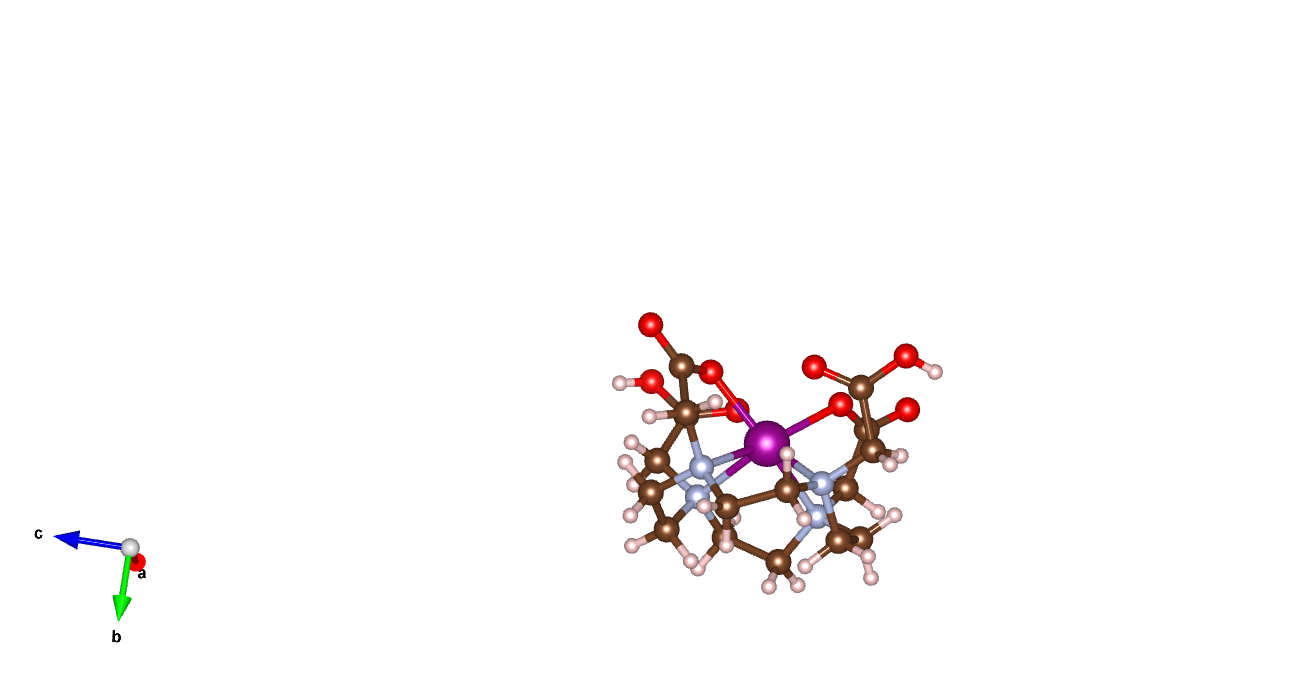
**

Determined crystal structure of DOTA head group complexed with Mn^2+^ in solid-state. Taken from CDCC ^[36]^

**References and Notes**

[30] B. Jagadish, G. L. Brickert-Albrecht, G. S. Nichol, E. A. Mash, N. Raghunand, *Tetrahedron Letters* **2011**, *52*, 2058–2061.

[31] N. Srinivasan, A. Yurek-George, A. Ganesan, *Mol Divers* **2005**, *9*, 291–293.

[32] Joos, A.; Rümenapp, C.; Wagner, F. E.; Gleich, B. Characterisation of Iron Oxide Nanoparticles by Mössbauer Spectroscopy at Ambient Temperature. *J. Magn. Magn. Mater.* **2016**, *399*, 123–129. https://doi.org/10.1016/j.jmmm.2015.09.060.

[33] M. K. C. T. Nagata, P. S. Brauchle, S. Wang, S. K. Briggs, Y. S. Hong, D. W. Laorenza, A. G. Lee, T. D. Westmoreland, *Polyhedron* **2016**, *114*, 299–305.

[34] Majumder, M.; Sathyamurthy, N. A Theoretical Investigation on the Effect of π–π Stacking Interaction on 1H Isotropic Chemical Shielding in Certain Homo- and Hetero-Nuclear Aromatic Systems. *Theor Chem Acc* **2012**, *131* (2), 1092. https://doi.org/10.1007/s00214-012-1092-3.

[35] Salikolimi, K.; Kawamoto, M.; He, P.; Aigaki, T.; Ito, Y. Polythiophene Nanoparticles That Display Reversible Multichromism in Aqueous Media. *Polym J* **2017**, *49* (5), 429–437. https://doi.org/10.1038/pj.2017.5.

[36] CCDC Mn_HONYUF contains the supplementary crystallographic data for this paper. These data can be obtained free of charge from The Cambridge Crystallographic Data Centre.
